# Reply: Absence of evidence is not evidence of absence for first trimester dydrogesterone-induced birth defects

**DOI:** 10.1093/hropen/hoae031

**Published:** 2024-05-11

**Authors:** Alexander Katalinic

**Affiliations:** Institute for Social Medicine and Epidemiology, University of Luebeck, Luebeck, Germany

Sir,

We would like to thank Quadros *et al.* (2024) for their letter regarding our article and also wish to thank you for the opportunity to comment on the points that they have raised.

In their letter, Quadros *et al.* expressed concerns regarding the quality, number of cases, and outcome assessment of the studies included in our systematic review (Katalinic *et al.*, 2024). They also commented on the inclusion of three additional studies for estimating the prevalence rate associated with dydrogesterone use in the first trimester of pregnancy. They then report the results of an analysis of pharmacovigilance data that they believe shows an association between dydrogesterone and congenital anomalies and suggest that we have overlooked this work. They conclude that our meta-analysis is not robust enough to draw conclusions regarding the safety of dydrogesterone (Quadros *et al.*, 2024).

First of all, it is possible that Quadros *et al.* are not familiar with the methodological procedures of a systematic review and the assessment of the results of such a review. The aim of a systematic review is to identify, critically review, and summarize all available evidence on a clearly defined question in a very standardized way, based on a predefined and registered study protocol using up-to-date methodology (Higgins *et al.*, 2023). This is what we have done. In their letter, Quadros *et al.* overlook two important aspects. First, the evidence summarized in our systematic review represents the currently best available and valid evidence on dydrogesterone exposure in the first trimester. Flawed, retracted, and critical biased publications, which can be found in the vastness of the internet, were excluded using a clear and reproducible methodology. Even if included, individual studies still have limitations: these limitations were clearly identified, assessed, and reported in a standardized manner. Some of the included studies have serious, but non-critical limitations, and could therefore be considered for the review. Subgroup analyses of high- and low-quality studies led to comparable results, and heterogeneity overall was very low. Therefore, our conclusions can be described as very robust. Second, it must be considered that the main finding of our systematic review was evaluated with regard to safety/uncertainty using the GRADE system. We clearly state that the evidence level for our findings was graded as low (leaving uncertainty), which is also reflected by the confidence interval for the main effect (three congenital anomalies fewer under dydrogesterone exposure; from 17 fewer to 21 more; based on 6 studies with 1512 children).

The assumption that we have overlooked the analysis by Henry *et al.* (2023) is inaccurate for several reasons. It is stated in the ‘Methods’ section of our article that the literature search extended to September 2022. The analysis in question by Henry *et al.* is from September 2023, is a half-page congress abstract and has not yet been published in a peer-reviewed format—therefore, it was impossible to include it. Even if this contribution had been eligible for review, it could not have been considered for our meta-analysis owing to a lack of information and the inability to critically evaluate the report according to review guidelines. The abstract leaves many questions open, as well as possible biases since reporting or detection bias cannot be ruled out (Matsuda *et al.*, 2015; Faillie, 2019).

We have already made it clear in our article that pharmacovigilance data can play an important role as a signal giver. However, the results of such analyses should only be interpreted as a sign and not more, because they may just as well be noise or prone to bias. Moreover, results based on pharmacovigilance data alone do not allow any causal conclusions to be drawn, especially if biological plausibility is lacking. Signs must be verified by adequate studies. In the case of dydrogesterone, such studies, which are superior to pharmacovigilance data, are already available and formed the basis of our systematic review.

We do not understand Quadros *et al.*’s (2024) objection to the estimation of the prevalence rate of congenital anomalies in dydrogesterone exposure. In order to make the estimate more robust, three cohort studies were included that were judged to be of sufficient quality, and in which all children were exposed to dydrogesterone. The pooled prevalence for congenital anomalies was 2.5% [95% CI 1.5–4.3%], with no big differences in the subgroups analyzed (RCT only: 3.4%, controlled cohort studies: 2.3%, and 1.8% in the three added studies; all with overlapping confidence limits).

To summarize, we disagree with Quadros *et al.* (2024) on these essential points. Our systematic review represents the best currently available evidence on the question of an increased rate of congenital anomalies after exposure to dydrogesterone in the first trimester. Based on the originally used PubMed/Medline search, no new studies could be identified at the time of writing (performed on 2 April 2024). A significant increase or decrease in the rate of congenital anomalies after dydrogesterone exposure was not detectable. As already discussed in our article (Katalinic *et al.*, 2024), while there may be a difference in rates of congenital anomalies, based on the confidence interval of the main finding of our systematic review, the difference would presumably be small and open in terms of which direction.
